# TRAIL and its receptors in cardiac diseases

**DOI:** 10.3389/fphys.2023.1256852

**Published:** 2023-08-09

**Authors:** Laurel A. Grisanti

**Affiliations:** Department of Biomedical Sciences, College of Veterinary Medicine, University of Missouri, Columbia, MO, United States

**Keywords:** TNF-related apoptosis inducing ligand, death receptors, cardiac disease, apoptosis, death receptor 5, extrinsic apoptosis, heart

## Abstract

Cardiovascular disease is a leading cause of death worldwide. Loss of cardiomyocytes that occurs during many types of damage to the heart such as ischemic injury and stress caused by pressure overload, diminishes cardiac function due to their limited regenerative capacity and promotes remodeling, which further damages the heart. Cardiomyocyte death occurs through two primary mechanisms, necrosis and apoptosis. Apoptosis is a highly regulated form of cell death that can occur through intrinsic (mitochondrial) or extrinsic (receptor mediated) pathways. Extrinsic apoptosis occurs through a subset of Tumor Necrosis Receptor (TNF) family receptors termed “Death Receptors.” While some ligands for death receptors have been extensively studied in the heart, such as TNF-α, others have been virtually unstudied. One poorly characterized cardiac TNF related ligand is TNF-Related Apoptosis Inducing Ligand (TRAIL). TRAIL binds to two apoptosis-inducing receptors, Death Receptor (DR) 4 and DR5. There are also three decoy TRAIL receptors, Decoy Receptor (DcR) 1, DcR2 and osteoprotegerin (OPG). While TRAIL has been extensively studied in the cancer field due to its ability to selectively induce apoptosis in transformed cell types, emerging clinical evidence points towards a role for TRAIL and its receptors in cardiac pathology. This article will highlight our current understanding of TRAIL and its receptors in normal and pathological conditions in the heart.

## Introduction

Cardiovascular disease is a significant healthcare problem and leading cause of death worldwide (Vaduganathan et al., 2022). Loss of cardiomyocytes through cell death mechanisms is a hallmark in the pathogenesis of many cardiac diseases including myocardial infarction and heart failure (Mishra et al., 2019). Cardiomyocytes lack the ability to substantially regenerate therefore, loss of cardiomyocytes results in a loss of contractile tissue and often leads to activation of remodeling pathways to form extracellular matrix scars resulting in further dysfunction (Eschenhagen et al., 2017; Thomas and Grisanti, 2020). Historically, cardiomyocyte death was thought to occur through two primary mechanisms, apoptosis and necrosis, that differ in their mechanisms and outcomes (Mishra et al., 2019). Recently, other forms of cardiomyocyte death including autophagy, pyroptosis, necroptosis and ferroptosis have been identified. A main difference between cell death mechanisms such as apoptosis and autophagy is that membrane integrity is maintained whereas in necrosis, necroptosis, ferroptosis and pyroptosis there is disruption of the cell membrane. With an intact plasma membrane, the dying cell becomes phagocytosed, resulting in minimal damage to the surrounding cells. Contrarily, release of cytoplasmic contents leads to the activation of pro-inflammatory mechanisms that further damage the tissue. Apoptosis has been the best studied form of cell death in the heart due to the long-held belief that it is a highly regulated, gene-directed process whereas necrosis has been thought to be an uncontrolled form of cell death however, emerging evidence suggests that the is not the case (Zhu and Sun, 2018). While all forms of cardiomyocyte death contribute to the pathogenesis of cardiac disease, it is widely understood and appreciated that apoptotic death of cardiomyocytes in adults is important in numerous myocardial diseases and may precede other forms of cardiomyocyte death such as necrosis (Kang and Izumo, 2000; Del Re et al., 2019).

Apoptosis occurs through two primary mechanisms, the intrinsic or mitochondrial and extrinsic or receptor-mediated pathways (Del Re et al., 2019). The intrinsic pathway is activated by various factors such as cellular stress, hypoxia and DNA damage. It has been extensively studied in the heart and relies on mitochondrial pathways. The extrinsic pathway occurs through activation of cell surface death receptors by extracellular ligands (Nagata, 1997). These receptors, termed “Death Receptors,” are a subset of the TNF receptor superfamily that contain an intracellular death domain (Ashkenazi and Dixit, 1998). Initiation of death receptor signaling occurs through various ligands belonging to the tumor necrosis (TNF) family. Some extrinsic pathways, such as the TNF-α ligand/receptor system, have been extensively characterized while others have been virtually unstudied in the context of cardiovascular disease and the heart (Amgalan et al., 2017; Schumacher and Naga Prasad, 2018).

## Death receptors

One member of the TNF family that is poorly understood in the context of the heart is TNF-Related Apoptosis-Inducing Ligand (TRAIL; also called TNFSF10, APO2L and CD253). TRAIL was first discovered for its homology to other TNF family members and its ability to induce apoptosis in cancer cells without affecting non-transformed cell populations (Wiley et al., 1995; Pitti et al., 1996). TRAIL is a type II transmembrane protein that can be cleaved from the cell surface to form a soluble ligand (Wiley et al., 1995). Membrane bound TRAIL is cleaved by cysteine proteases, such as matrix metalloproteinases (MMPs) and a disintegrin and metalloproteinases (ADAMs), to release soluble TRAIL, which then can enter the circulation to be distributed throughout the body (Wajant et al., 2001; Kimberley and Screaton, 2004). The exact protease responsible for TRAIL cleavage is unclear. *In vivo* studies have not been performed and *in vitro* studies have been controversial with MMP inhibitors being found to both prevent cleavage and have no impact on cleavage depending on the study (Naval et al., 2019). Both soluble and membrane bound TRAIL are thought to be capable of activating TRAIL receptors however, differences in their efficacy may occur (Wajant et al., 2001; Kimberley and Screaton, 2004). Membrane TRAIL is thought to be a more potent agonist due to the enhanced ability to aggregate and thus become more efficient at receptor activation (Wajant et al., 2001; Kimberley and Screaton, 2004). Additionally, differences in the location of their effects may occur between soluble and membrane TRAIL since soluble TRAIL is capable of entering the circulation and being distributed systemically whereas membrane bound TRAIL is restricted to binding to TRAIL receptors on adjacent cells. While many cell populations are capable of TRAIL generation, myeloid cell populations, such as neutrophils and monocytes, are thought to be a large source of circulating TRAIL (Tecchio et al., 2004; Falschlehner et al., 2009). The ability of these cells to migrate throughout the body may aid with efficient TRAIL receptor activation and indeed, factors that enhance TRAIL generation and cleavage include inflammatory cytokines amongst other factors, further suggesting that immune cells are an important source of TRAIL generation. Activation of TRAIL receptors requires the formation of a TRAIL homotrimer that binds to a cluster of three receptors to activate downstream signaling.

**FIGURE 1:**
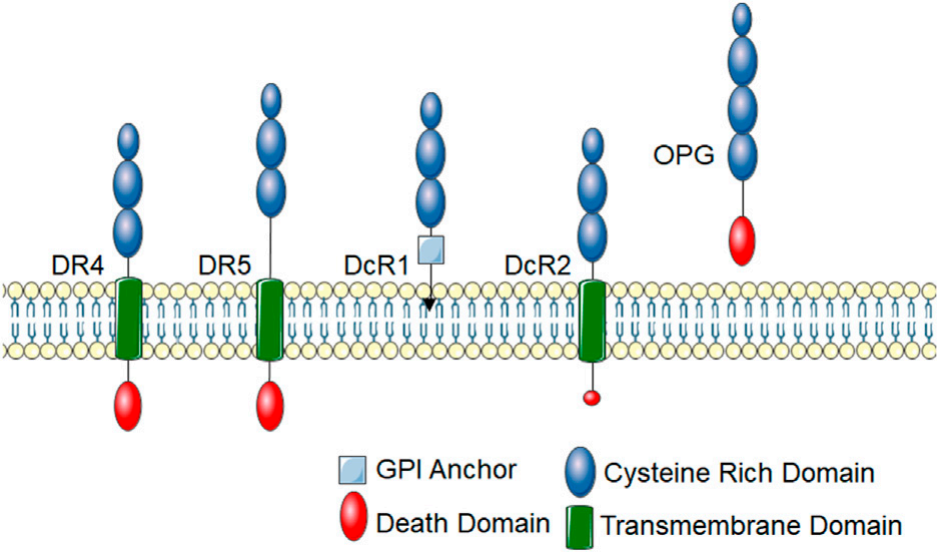
TRAIL receptors. TRAIL receptors belong to the TNF super family and share typical homology to other TNF superfamily receptors. These receptors have an extracellular cysteine-rich domain. There are five identified TRAIL receptors. DR4 and DR5 are death-inducing receptors that have an intracellular protein-protein interaction domain called a death domain, which links them to cytotoxic signaling cascades. DcR1 contains a GPI-anchor and lacks an intracellular domain, thus cannot initiate intracellular signal transduction cascades. DcR2 has a truncated death domain that renders it non-function in recruiting cytotoxic signaling molecules. OPG is a soluble TRAIL receptor that also binds RANKL.

In humans, TRAIL binds to two death inducing receptors, Death Receptor (DR) 4 (also called TRAIL-R1 and TNFRSF10A) and DR5 (also called TRAIL-R2 and TNFRSF10B) (Pan et al., 1997a; Walczak et al., 1997). Like all TNF receptor superfamily members, DR4 and DR5 have an extracellular cysteine-rich domain. Death inducing receptors, such as DR4 and DR5, have an intracellular death domain that allows for protein-protein interactions to initiate cytotoxic downstream signaling events (Figure 1). There are also two decoy TRAIL receptors, DcR1 (also called TNFRSF10C and TRAIL-R3), which lacks the cytosolic domain, and DcR2 (also called TNFRSF10D and TRAIL-R4), which lacks the death domain (Pan et al., 1997b; Pan et al., 1998). DcR1 lacks a membrane spanning domain and intracellular death domain and is anchored to the membrane with a glycosylphosphatidylinositol (GPI) anchor. DcR2 has a truncated death domain that seems to make it non-function in initiating cell death signaling. Canonical DR4 and DR5 signaling leads to the activation of the extrinsic apoptosis pathway and activation of caspase 8, which activate the effector caspases, caspase 3 or 7. DcR1 and DcR2 have historically been thought to inhibit TRAIL signaling by acting as decoys to prevent TRAIL binding to DR4 or DR5, however, emerging evidence suggests that they may have biological functions. Of note, through protein interactions in its truncated death domain, DcR2 can activate alternative signaling pathways such as NF-κB (Yang et al., 2018). Additionally, osteoprotegerin (OPG; also called osteoclastogenesis inhibitory factor (OCIF) or TNFRSF11B) can act as a soluble TRAIL decoy receptor (Emery et al., 1998; Miyashita et al., 2004). OPG is a soluble glycoprotein that belongs to the TNF receptor family. It was originally identified for its role in receptor activator of nuclear factor kappa-B (RANK)/RANK ligand (RANKL) signaling and described as a decoy receptor for RANKL (Emery et al., 1998; Miyashita et al., 2004). RANKL is the main regulator of osteoclast biology and also plays an important role in immune function (Yasuda et al., 1998). While OPG contains a death domain, it is not intracellular and thus does not initiate apoptotic signaling cascades. Though TRAIL binds to multiple receptors, there are substantial differences in its affinity to these TRAIL receptors. Under physiological conditions, TRAIL is thought to have the highest binding affinity for DR5 with DR4 being 30-fold lower, decoy receptors 100-fold lower and OPG over 200-fold lower affinity (Truneh et al., 2000). A further mechanism of regulation for TRAIL signaling is that, like many members of the TNFR superfamily, there is evidence that DR4 and DR5 can be cleaved by proteases to release the extracellular portion of the receptor (Levine et al., 2005; Vunnam et al., 2017). This soluble form of the receptor lacks the intracellular death domain and thus cannot illicit signaling. Indeed, these soluble receptors may act as stronger decoy receptors than DcR1, DcR2 and OPG due to their high affinity for TRAIL (Vunnam et al., 2017). Though the mechanisms of extracellular cleavage and the impact soluble receptors have on TRAIL signaling have not been thoroughly examined for TRAIL receptors, these soluble receptors likely play an important role in physiology and their levels have become important biomarkers for disease, often correlating with inhibition of TRAIL receptor signaling (Yasuda et al., 1998; Wan et al., 2019).

**FIGURE 2:**
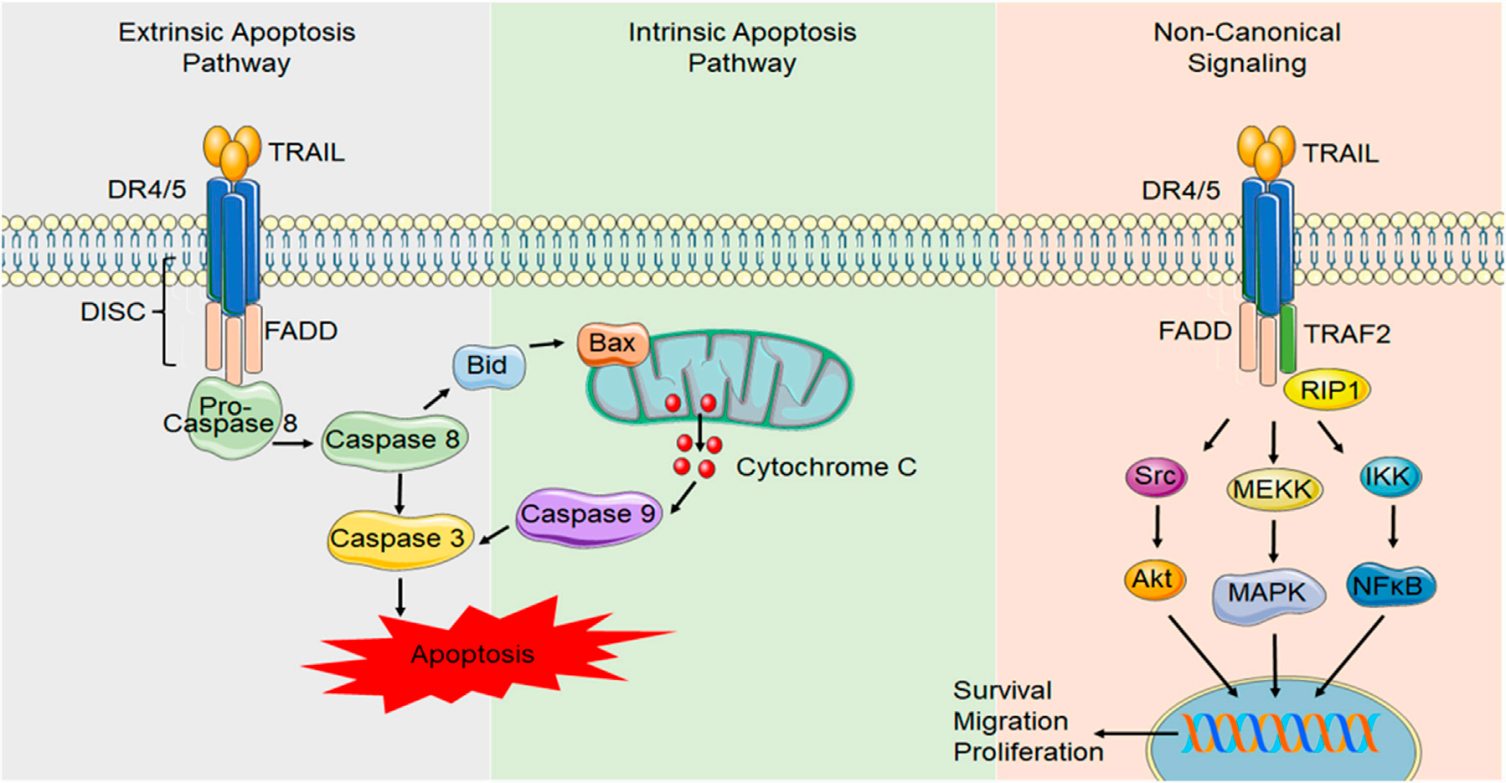
Canonical and non-canonical DR4/5 signaling. In canonical DR signaling, ligand binding to the death domain containing TRAIL receptors, DR4 and DR5, causes receptor trimerization and results in the formation of a DISC. DISC formation allows FADD to activate the initiator caspase, caspase 8. Caspase 8 can activate initiator caspases, such as caspase 3, which induces apoptosis. Alternatively, caspase 8 can activate the intrinsic/mitochondrial mechanism of apoptosis through the activation of Bid. During TRAIL resistance or in non-transformed cell types, non-canonical TRAIL signaling occurs NF-κB signaling can be initiated through FADD recruitment and caspase 8 activation. Alternatively, other proteins such as receptor-interacting protein (RIP) can compete with FADD binding and allow TNF receptor-associated factor 2 (TRAF2) to bind, initiating other signals such as Akt, the mitogen activated protein kinases (MAPK) such as ERK1/2 and NF-κB signaling that can initiate transcription of pro-survival, migratory and proliferative genes.

For receptor binding, TRAIL forms a homotrimer that binds to high affinity death-inducing receptors, DR4 and DR5, resulting in trimerization of the receptor, causing the formation of a death-inducing signaling complex (DISC), that can then recruit the adapter protein Fas-associated death domain (FADD), which acts as an intermediate between the receptor and the pro-domain of the initiator caspase, caspase 8 (Wiley et al., 1995) (Figure 2). Dimerization of caspase 8 leads to the formation of mature caspase 8, which then activates effector caspases such as caspase 3, resulting in cell apoptosis. In some cases, this process can be enhanced by activation of additional, mitochondrial apoptosis pathways (Lu et al., 2014).

## TRAIL and its receptors in the heart

Since TRAIL was first discovered for its ability to induce apoptosis in cancer cells without affecting non-transformed cell populations (Wiley et al., 1995; Pitti et al., 1996), TRAIL receptors, in particular DR5, have been an attractive therapeutic target for cancer over the past 30 years (von Karstedt et al., 2017). Though the role of TRAIL has been extensively studied in transformed cells and its ability to initiate apoptosis through classical death receptor pathways is well known, its role in normal cells has been largely ignored. Phase I clinical trials have identified no adverse effects of DR5 agonist administration further supporting a non-death-inducing role in non-cancerous tissues (Ashkenazi et al., 1999; Walczak et al., 1999; Herbst et al., 2010; Soria et al., 2010). Further supporting this, mice lacking DR5 have no baseline phenotype with the exception of a reduced thymus size (Finnberg et al., 2005). TRAIL knockout animals also have no overt phenotype in the absence of disease but may have an increased susceptibility to spontaneous tumor metastasis (Cretney et al., 2002; Sedger et al., 2002; Lamhamedi-Cherradi et al., 2003). Mice lacking OPG have severe arterial calcification, but this has been attributed to its role in RANK/RANKL signaling (Bucay et al., 1998). This led to the longstanding belief that DR4 and DR5 did have a role in non-transformed cell populations. However, TRAIL is produced by most cell types and there is ubiquitous TRAIL receptor expression throughout the body where they importance is poorly understood (Pan et al., 1997a; Pan et al., 1997b; Pan et al., 1998; Tecchio et al., 2004; Falschlehner et al., 2009). TRAIL and its receptors are expressed in virtually all tissues of the body. Additionally, high expression of TRAIL and all four of its receptors have been reported at the transcript and protein level in the human heart (Pan et al., 1997a; Pan et al., 1997b; Ozoren and El-Deiry, 2003; Spierings et al., 2004) and DR5 expression is also present in the rodent heart were its function has been essentially unstudied (Wu et al., 1999; Ozoren and El-Deiry, 2003; Tanner et al., 2019).

It is widely appreciated that activation of DR4 and DR5 in the majority of healthy cells does not result in the induction of apoptosis and emerging evidence suggests it can lead to pro-survival pathway activation such as Extracellular-Regulated Kinase (ERK) 1/2, Akt and Nuclear Factor (NF)-κB (Benedict and Ware, 2012) (Figure 2). The mechanisms behind why transformed cells are susceptible to TRAIL-induced apoptosis while normal cells are not is currently unclear but it may be due to distribution of non-death-inducing TRAIL receptors, differences in the expression or localization of subcellular components or changes in post-translational modifications of the receptor (Leverkus et al., 2000; Hunter and Nixon, 2006; Song et al., 2007). These conditions are likely not permanent and may vary over time with changing physiological conditions, such as during disease states. Indeed, the ability of transformed cells to adapt and become resistant to TRAIL-induced cell death has posed a challenge and has been the main limiting factor when targeting DR4 and DR5 for cancer therapies. Trials targeting DR5 have been largely disappointing with little benefit on patient survival (Snajdauf et al., 2021). While this has been largely attributed to the ability of cancer cells to develop TRAIL resistance (Dimberg et al., 2013), this demonstrates our lack of understanding of TRAIL and its receptors. All TRAIL receptors can be glycosylated however, it is not currently clear the role glycosylation plays. N-glycosylation of murine DR5 and O-glycosylation of human DR5 has been shown to promote apoptotic signaling (Wagner et al., 2007; Dufour et al., 2017). However, other studies have demonstrated prevention of N-glycosylation of mouse DR5 enhances TRAIL binding and induction of cell death (Estornes et al., 2018). Palmitoylation has been shown to direct DR5 to lipid microdomains in the plasma membrane (Ko et al., 1999; Fritsch et al., 2021). This appears to be important for receptor clustering and DISC interactions to induce caspase 8 activation (Ko et al., 1999). Furthermore, alterations in DR expression, levels of intracellular signaling proteins such as caspase 8 and changes in decoy receptor expression can impact a cell’s response to TRAIL (Gomez-Benito et al., 2007; Azijli et al., 2013; Tanner and Grisanti, 2021). In light of these new developments in TRAIL signaling, more recent evidence is emerging suggesting a pleiotropic role of TRAIL in physiology. Emerging evidence points to a role for TRAIL and its receptors in numerous cellular processes including proliferation, migration and differentiation.

While TRAIL and its receptors have been implicated in cancer for decades, emerging evidence suggests a role in a multitude of different diseases including viral infection, arthritis, diabetes and cardiovascular disease (Wajant, 2019). Mice lacking TRAIL have increased susceptibility to experimental tumor metastasis (Sedger et al., 2002). However, they also have increased susceptibility to induction of autoimmune diseases including collagen-induced arthritis and streptozotocin-induced diabetes (Lamhamedi-Cherradi et al., 2003). These mice had severe deficiencies in thymocyte apoptosis resulting in impaired thymic deletion induced by T cell receptor ligation suggesting an important role for TRAIL in regulating inflammation. Of particular note, TRAIL has been linked to multiple risk factors of disease including hypercholesterolemia, smoking, diabetes and hypertension (Benedict and Ware, 2012; Cheng et al., 2015; di Giuseppe et al., 2017; Mattisson et al., 2017; Hameed et al., 2012). Recently, TRAIL, DR5 and OPG have been linked to multiple forms of human heart failure however, the role of TRAIL and its receptors is just emerging in the heart (Secchiero et al., 2009; Skau et al., 2017; Stenemo et al., 2018).

## Clinical studies

While TRAIL and its receptors have been implicated in cancer for decades, emerging evidence suggests a role in a multitude of different diseases including arthritis, diabetes and cardiovascular disease (Benedict and Ware, 2012). TRAIL and its receptors are associated with many cardiovascular risk factors including smoking, coronary artery disease (CAD) and diabetes (Benedict and Ware, 2012; Cheng et al., 2015; di Giuseppe et al., 2017; Mattisson et al., 2017; Hameed et al., 2012). OPG is also associated with age, smoking, diabetes, C-reactive protein, sex hormone-binding globulin and coronary heart disease in men and women (di Giuseppe et al., 2017). In the past few years, TRAIL or its receptors have been associated with multiple cardiac diseases however, the role of TRAIL and its receptors is just emerging in the heart (Table 1) (Secchiero et al., 2009; Skau et al., 2017; Stenemo et al., 2018). In a study examining the relationship between circulating TRAIL and all cause cardiovascular disease in elderly patients, TRAIL was found to strongly correlate with mortality due to cardiovascular disease (Volpato et al., 2011). Patients were monitored over 6 years following baseline plasma TRAIL assessment. Interestingly, TRAIL levels were inversely related to all-cause mortality, with TRAIL being particularly strongly correlated with patients succumbing to cardiovascular disease with it being lesser associated with mortality due to other causes. This points to a potentially protective role for TRAIL in cardiovascular disease.

**TABLE 1:** A summary of human studies identifying a relationship between TRAIL and its receptors and cardiac disease.

Patient Population	Study Type	Outcome	Relationship with Disease	Citation
All Cause Cardiovascular Disease				
1,282 older patients with cardiovascular disease	Prospective observational; 6 years follow-up	Association between prevalent cardiovascular disease and TRAIL	Inverse correlation	Volpato et al., (2011)
Arrhythmias				
25 patients with AF	Prospective observational; Before epicardial ablation with 3 and 6 months follow-up	Serum TRAIL decreased with successful ablation	Positive correlation	Osmancik et al., (2010)
45 patients with AF with cardioversion	Prospective observational; 6 months follow-up	TRAIL transcardiac gradient (coronary sinus concentration-aortic root concentration) was a negative predictor of AF recurrence	Inverse correlation	Deftereos et al., (2013)
60 patients with acute onset AF	Prospective observational; Initial measurement and 7–10 days after pharmacological cardioversion	Presence of cardiovascular comorbidities as connected with higher OPG and lower TRAIL initially with AF, increased TRAIL in patients with sinus rhythm maintenance	Positive correlation	Rewiuk and Grodzicki, (2015)
638 patients (62% male) with AF	Prospective observational	40 common biomarkers identified decreased soluble DR5 as associated with AF incidence	Inverse correlation	Chua et al., (2019)
Coronary Artery Disease				
85 patients (72% male) after percutaneous coronary intervention	Prospective Observational	Higher soluble TRAIL levels in patients with CAD. TRAIL levels remained elevated in patients without restenosis	Positive correlation	Satoh et al., (2010)
285 patients (79% male) undergoing coronary angiography	Cross-sectional study	Serum TRAIL levels were lower in patients with CAD and associated with severity	Inverse correlation	Mori et al., (2010)
Acute Myocardial Infarction				
60 control and 60 acute myocardial infarction patients	Case-controlled study; Acute and 1 year follow-up	Serum TRAIL decreased acutely, low TRAIL levels were associated with increased incidence of cardiac death and heart failure	Inverse correlation	Secchiero et al., (2009)
113 patients with acute myocardial infarction, 21 with unstable angina and 120 healthy controls	Case-controlled study	TRAIL is decreased with acute myocardial infarction, CAD patients had an increased OPG/TRAIL ratio	Inverse correlation	Secchiero et al., (2010)
28 patients with acute myocardial infarction, 32 with stable CAD and 20 healthy controls	Case-controlled study	Acute myocardial infarction had elevated OPG and lower TRAIL compared with stable CAD and controls	Inverse correlation	Shaker et al., (2010)
847 patients (67.3% male) with acute myocardial infarction	Prospective observational; all cause mortality as the end point	Proteomic profiling identified soluble DR5 as an independent predictor of long-term all cause mortality	Inverse correlation	Skau et al., (2017)
101 patients with ST-elevation myocardial infarction treated with percutaneous coronary intervention	Prospective observational; 1 month and 2 year follow-up	Serum TRAIL decreased 1 day after percutaneous coronary intervention followed by an increase to 1 month. Low TRAIL was associated with worse ejection fraction	Inverse correlation	Teringova et al., (2018)
Chronic Heart Failure				
86 control and 105 male patients with non-ischemic cardiomyopathy	Case-controlled study	Plasma TRAIL were elevated and OPG levels did not change. TRAIL was positively correlated with LVEDD. TRAIL was increased in PBLs whereas OPG was upregulated in endomyocardial samples	Positive correlation	Schoppet et al., (2005)
350 patients (66% male)	Case-controlled study; 2 years follow-up	Low TRAIL levels were associated with risk of rehospitalization and death	Inverse correlation	Niessner et al., (2009)
Elder male (71%) and females of European descent without heart failure at baseline	Prospective observational; up to 11 years follow-up	Proteomic profiling identified soluble DR5 and OPG as risk factors for heart failure development	Inverse correlation	Stenemo et al., (2018)
Heart Failure with Preserved Ejection Fraction				
86 (49% male) patients with LVEF ≥45% and pro-NT-BNP >300 ng/L	Prospective observational	Plasma TRAIL was identified as negatively and soluble DR5 was positively associated with outcome	Inverse correlation	Hage et al., (2017)
Myocarditis				
31 males and females with Chagas cardiomyopathy	Case-controlled study	Plasma TRAIL was elevated with severe disease	Positive correlation	Lula et al., (2009)

There have been several human studies investigating the role of TRAIL and its receptors in specific types of cardiac disease. However, many of these studies have a low number and often homogenous population of patients. Nevertheless, TRAIL, DR5 and OPG are emerging as potential predictive markers, biomarkers and relevant in the study of cardiac diseases.

### Arrhythmias

Arrhythmias arise from disrupted electrical conduction in the heart. Changes in conduction promote apoptosis, fibrosis and remodeling in the heart, which potentiates the disease and can lead to heart failure development (Grisanti, 2018) This remodeling that occurs with arrhythmias brought interest to the contribution of TRAIL and its receptors in the setting of cardiac arrhythmias. In a study looking at markers of apoptosis in atrial fibrillation (AF) patients, serum markers of apoptosis, including TRAIL, were decreased with successful epicardial ablation whereas TRAIL levels did not change in patients with reoccurring AF (Osmancik et al, 2010). In AF patients undergoing cardioversion, blood samples obtained from the coronary sinus and aortic root showed a transcardiac gradient in soluble TRAIL whereas other parameters including interleukin (IL)-6 and C-reactive protein, did not (Deftereos et al, 2013). Additionally, this study found no difference in peripheral TRAIL levels. In this study, the TRAIL gradient was inversely associated with AF recurrence, suggesting a protective role. With acute onset AF, patients with cardiovascular comorbidities had higher circulating OPG and lower TRAIL (Rewiuk and Grodicki, 2015). Restoration of sinus rhythm increased TRAIL concentrations in patients with sinus rhythm maintenance. In a study examining 40 common cardiovascular biomarkers in patients with known AF, soluble DR5 was amongst several markers associated with AF (Chua et al, 2019). Since soluble DR5 lacks the intracellular domain necessary to illicit signaling, circulating DR5 levels are thought to be inversely related to DR5 activation at the tissue level. In this study reduced soluble DR5 was associated with AF suggesting enhanced DR5 signaling in the tissue.

### Atherosclerosis

Atherosclerosis develops in the walls of arteries causing narrowing of blood vessels and limiting blood supply to parts of the body. This is particularly important in the heart, where CAD limits oxygen and nutrient supply to the heart and can result in myocardial infarction. In a group of diabetic patients with early-stage atherosclerosis, TRAIL was not associated with carotid or femoral intima-media thickness (Kawano et al., 2011). In patients with chronic kidney disease, low circulating TRAIL levels were associated with the appearance of new atheromatous plaques in a 24 months follow-up study (Arcidiacono et al., 2018). However, TRAIL levels measured from the coronary artery of patients with stable angina or positive ischemia noninvasive test showed an inverse association with TRAIL levels in the necrotic core and fibrofatty content of atheromatous plaques also had decreased TRAIL levels, though to a lesser degree (Deftereos et al., 2012). Plasma TRAIL levels have been shown to be elevated systemically in patients with CAD (Satoh et al., 2010). However, other studies observe decreased TRAIL levels in patients with CAD that are associated with severity (Mori et al., 2010). Furthermore, OPG serum levels have been shown to be increased in CAD and are associated with cardiovascular mortality (Collin-Osdoby, 2004).

### Acute myocardial infarction

Myocardial infarction arises when blood flow is limited or stops in the coronary artery of the heart leading to damaged heart muscle. There have been several studies associating TRAIL and its receptors with acute myocardial infarction (AMI). High soluble DR5 and TRAIL in atherosclerotic plaques have been found to be associated with increased risk of future cardiovascular events (Goncalves et al., 2019). Several studies have shown that serum TRAIL is downregulated in AMI patients (Skau et al., 2017; Wang et al., 2020). In a study examining the role of OPG and TRAIL in CAD, patients with AMI and CAD had higher levels of circulating OPG and lower levels of TRAIL compared to patients with stable CAD or healthy controls, which was aggravated as the number of affected coronary vessels increased (Shaker et al., 2010). Other studies have shown that OPG is increased in patients with unstable angina and AMI while TRAIL only different with AMI where it was found to be decreased (Secchiero et al., 2010). In patients with ST-elevation myocardial infarction treated with primary percutaneous coronary intervention, serum TRAIL decreased 1 day after reperfusion, then was elevated 1 month after reperfusion (Teringova et al., 2018). Early TRAIL levels at 1 or 2 days after reperfusion showed an inverse correlation with troponin I and a positive correlation with left ventricular ejection fraction. One month following reperfusion, TRAIL was found to be correlated with ejection fraction. However, TRAIL was not able to predict major adverse cardiovascular events in a 2-year follow up.

Interestingly, TRAIL may be a powerful biomarker for myocardial infarction patients. In a study investigating 92 biomarkers linked to cardiovascular disease or inflammation to determine which biomarkers predicted long-term all-cause mortality in patients with AMI, growth differentiation factor (GDF)-15 and soluble DR5 were identified as the most powerful biomarkers in predicting long-term all-cause mortality (Skau et al., 2017). In this study, low levels of circulating DR5 were associated with worsened outcome. Additionally, an examination of TRAIL levels in patients with AMI showed serum TRAIL levels were inversely correlated with other prognostic markers for adverse cardiovascular events such as creatine kinase (CK) and brain natriuretic peptide (BNP) (Secchiero et al., 2009). This would suggest that decreased serum levels of TRAIL might be negative in terms of outcome after myocardial infarction. However, these changes are likely not specific to the heart since changes in circulating TRAIL are also associated with other ischemic events such as ischemic stroke (Stanne et al., 2022). Furthermore, therapies that protect the heart after myocardial infarction, such as postconditioning, appear to positively influence TRAIL (Luz et al., 2017). Interestingly, TRAIL expression is upregulated on peripheral blood mononuclear cells (PBMCs) of patients with AMI (Nakajima et al., 2003). This is localized primarily to macrophage and T cell populations and may suggest that immune cells are an important source of TRAIL in cardiovascular pathology.

### Heart failure

Heart failure can arise from several different causes including myocardial infarction, high blood pressure, arrythmias and infection and culminates in the heart not pumping enough blood to meet the needs of the body. Proteomic profiling of 80 proteins associated with cardiovascular pathology in elderly males looking at predictors of heart failure development over an 11-year span identified OPG and soluble DR5 as two of eighteen proteins as being associated with heart failure incidence (Stenemo et al., 2018). In this study, high levels of circulating DR5 were associated with worsened systolic function. In a study examining Fas and TRAIL levels in the progression of heart failure, high soluble TRAIL concentrations were associated with better prognosis, which occurred regardless of heart failure etiology (Niessner et al., 2009). When more specifically investigating dilated cardiomyopathy, TRAIL plasma concentrations were elevated in males with dilated cardiomyopathy and positively correlated with left ventricular end diastolic diameter (Schoppet et al., 2005). In this study, OPG plasma levels did not differ from controls. Both TRAIL and OPG protein were found in peripheral blood leukocytes (PBLs) and endomyocardial biopsy samples with changes in TRAIL occurring both in PBLs and myocardium whereas OPG was only different in the heart. This suggests that TRAIL upregulation occurs systemically whereas OPG changes occur locally. However, other studies have also found changes in circulating OPG in patients with chronic heart failure. High baseline OPG levels have been strongly associated with incidence of death and found to be a significant predictor of death (Roysland et al., 2010). OPG is also elevated in heart failure and after AMI (Collin-Osdoby, 2004; Ueland et al., 2004). However, it is currently unclear the importance of OPG in regulating TRAIL binding in cardiac diseases. TRAIL binding to DR5 occurs at a much higher affinity than OPG (Truneh et al., 2000). Currently, it is unknown if TRAIL levels are high enough for OPG to play significant role in sequestering TRAIL. In patients that developed heart failure after myocardial infarction, OPG levels were elevated and TRAIL levels reduced compared to patients that did not develop heart failure (Secchiero et al., 2010). These studies suggest that the ratio between OPG and TRAIL may be a useful marker for determining a patient’s risk for development heart failure after myocardial infarction. Additionally, other studies suggest that the OPG/TRAIL ratio may be an important indicator for heart failure risk regardless of etiology (Toffoli et al., 2012). TRAIL and its receptors have also been implicated in other forms of heart failure. In a study looking at genes associated with prognosis in heart failure with preserved ejection fraction (HFpEF), TRAIL was the most gene most negatively associated with outcome whereas soluble DR5 was positively associated with outcome (Hage et al., 2017). Lower levels of the ligand and increased levels of decoy receptors were associated with worsened outcome suggesting a beneficial role of TRAIL signaling.

The extrinsic pathway of apoptosis is known to play an important role in the development of myocarditis-induced cardiac dysfunction. Myocarditis arises as a result of viral, bacterial, toxin, autoimmune and other causes of inflammation. As with other forms of heart failure, myocarditis can result in cardiomyocytes death through multiple forms of cell death including the extrinsic pathway of apoptosis (DeBiasi et al., 2010). Very few studies have investigated the role of TRAIL and its receptors in myocarditis however, in Chagas cardiomyopathy, plasma levels of TRAIL and other TNF superfamily ligands are elevated, particularly with severe disease, suggesting a detrimental role for TRAIL (Lula et al., 2009). Due to the importance of TRAIL in inflammation and the immune system and the connection between inflammation and myocarditis, it would not be surprising if TRAIL and its receptors are found to play an important role in disease progression in myocarditis and have a role in myocarditis cause by other etiologies.

Taken together, these clinical studies have identified changes in TRAIL or its receptors in a number of cardiac pathologies including arrhythmias, coronary artery disease, myocardial infarction, heart failure and myocarditis. However, the findings from these studies are often contradictory with some studies showing a positive association with TRAIL and its receptors with disease severity while others show an inverse correlation. Many of these studies have a limited sample size and often lack a diverse patient population. Large scale, carefully controlled studies will be necessary to decisively identify the role of TRAIL and its receptors in cardiac disease. Additionally, it is important to consider that the contribution of TRAIL and its receptors may differ depending on the type of cardiac disease. In some instances, TRAIL may serve a pro-apoptotic function in cardiomyocytes and exacerbate cardiac dysfunction whereas it in other cases it may be limiting inflammation and beneficial to disease pathology. Future mechanistic studies will be necessary to determine the role of the TRAIL receptor system in various cardiac pathologies.

### Preclinical studies

With the growing abundance of human evidence suggesting the involvement of TRAIL and its receptors in multiple forms of cardiovascular disease and heart failure from several etiologies, it is surprising that our understanding of the role of TRAIL and its receptors in the heart is limited. The majority of human studies have investigated circulating factors such as TRAIL, soluble DR5 or OPG, with little direct evidence of a localized role for TRAIL signaling in the heart. However, there is emerging evidence that supports direct role in regulating cardiac function and pathophysiology.

DR4 and DR5 have been shown to be expressed in the healthy heart (Nakajima et al., 2003). However, TRAIL and its receptors appear to be dynamic and undergo changes during disease states. During many disease states including heart failure, increased sympathetic activity occurs and contributes to disease pathology. Cardiac TRAIL alterations have been associated with β-adrenergic receptor agonist administration in mice (Grisanti et al., 2014). Similarly, TRAIL is upregulated in the myocardium of rats with experimental myocardial infarction (Wang et al., 2020) and patients with dilated cardiomyopathy (Schoppet et al., 2005; Dzimiri et al., 2007). These findings for changes in DR5 in dilated cardiomyopathy myocardium have been confirmed in other studies, which also show upregulation of DR4 and with a converse downregulation of DcR1 and DcR2 (Dzimiri et al., 2007). DR5 transcript expression has been shown to be upregulated with β-adrenergic receptor agonists with acute administration in mice (Talarico et al., 2014) and in rat and human heart slices (Herrmann et al., 2014). In rats with experimental myocardial infarction, OPG was upregulated in the ischemic and non-ischemic portions of the heart, whereas RANK and RANKL were elevated strictly in the ischemic region (Ueland et al., 2005). These changes were attributed to the non-cardiomyocyte populations of the heart. Serum OPG was elevated in both ischemic and non-ischemic heart failure patients. Similarly, cardiac expression of OPG was increased in patients with heart failure.

The majority of studies investigating the role of TRAIL and its receptors in the heart have taken a systemic approach however, it is widely appreciated that the heart is composed of many cell types (Pinto et al., 2016; Zhou and Pu, 2016). While there is debate about the exact ratios of cells, which may differ between species, it is appreciated that cardiomyocytes make up 30%–40% of the cells, but the majority of the volume. Appreciable levels of fibroblasts, endothelial cells and immune cells are also present and play an important contribution in homeostasis and during pathology (Pinto et al., 2016; Zhou and Pu, 2016). Most evidence for cell type specific functions of TRAIL and its receptors in the heart is convoluted with often times contradictory reports. However, since each cell type has a unique role in cardiac function, future studies to understand the role of TRAIL and its receptors in relevant cell populations with help with understanding their importance in cardiac disease.

### Cardiomyocytes

**FIGURE 3:**
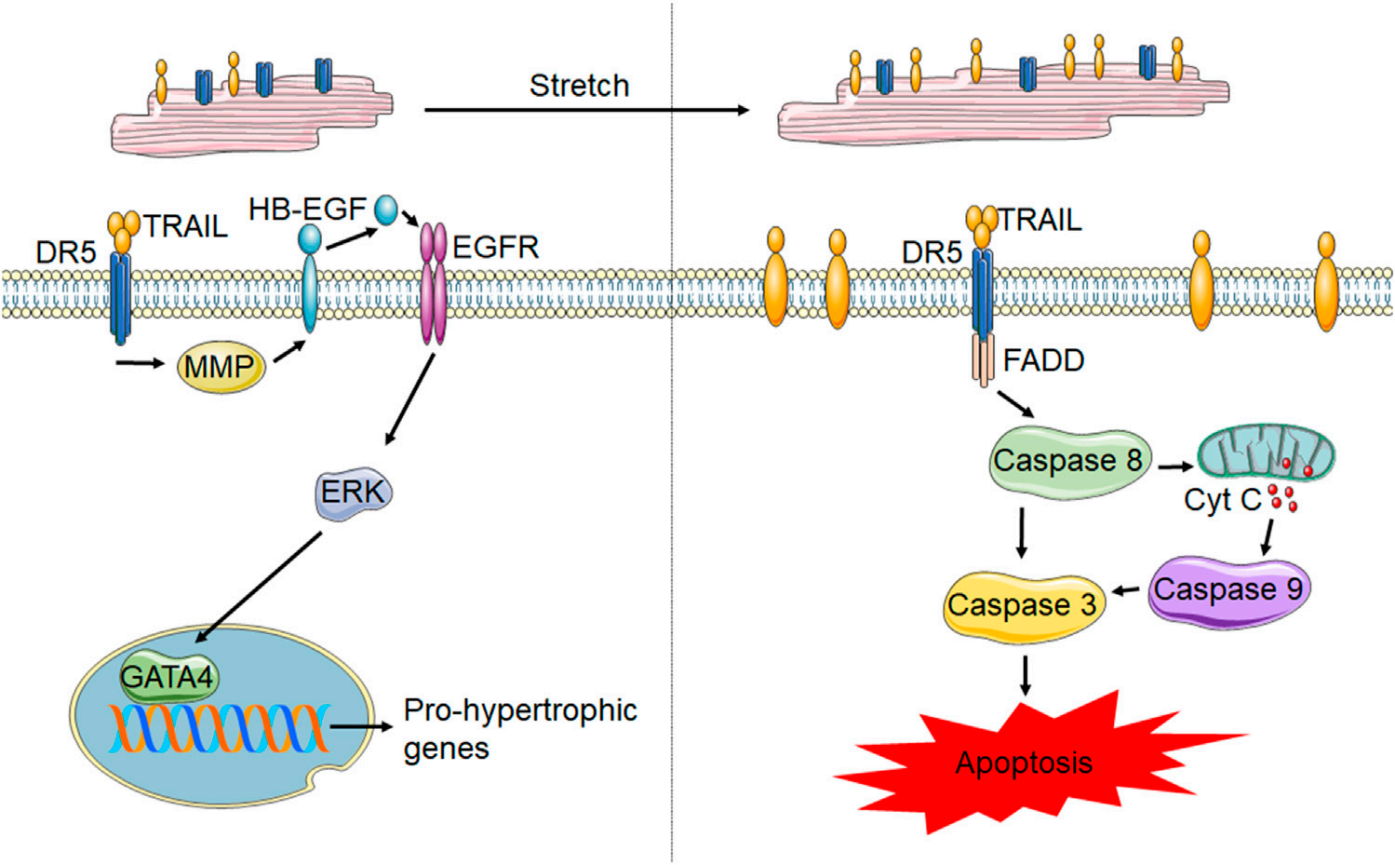
TRAIL and DR5 in cardiomyocytes. In healthy cardiomyocytes (left) activation of DR5 has been shown to increase pro-hypertrophic gene transcription through the MMP-dependent cleavage of HB-EGF, which activates EGFR. EGFR activation results in ERK1/2 phosphorylation and subsequent activation of the transcription factor, GATA4, resulting in gene transcription. However, following stretch and potentially other forms of cardiomyocyte stress, membrane TRAIL is upregulated, which leads to cardiomyocyte apoptosis through activation of the caspases 8, 9 and 3.

Information pertaining to the cell type specific roles of TRAIL and its receptors in the heart is limited. Multiple studies have identified TRAIL receptor expression on cardiomyocytes. Membrane localization of DR5 has been shown in rodent cardiomyocytes (Grisanti et al., 2014; Tanner et al., 2019) and mechanically stretched rat neonatal cardiomyocytes upregulate TRAIL expression (Liao et al., 2005). However, the role of TRAIL and its receptors in cardiomyocytes is not clear. A combination of positively and negatively correlated TRAIL levels with cardiac diseases could indicate either a pro- or anti-apoptotic role. Additionally, the alterations in TRAIL and its receptors systemically could not be indicative of what is happening locally in the heart. Secretion of TRAIL by cardiomyocytes suggests a role for localized signaling in the heart (Liao et al., 2005; Grisanti et al., 2014). However, studies from isolated cardiomyocytes have been conflicting. Neonatal rat cardiomyocytes subjected to stretch are susceptible to TRAIL-mediated apoptosis whereas inactivation of TRAIL by soluble DR5 neutralized stretch-induced apoptosis (Liao et al., 2005). However, other studies have shown no impact of TRAIL or DR5 agonists in apoptosis activation in neonatal rat cardiomyocytes (Tanner et al., 2019). Supporting this, DR5 knockout mice have no alterations in cardiomyocyte death in response to chronic β-adrenergic receptor stimulation (Tanner and Grisanti, 2021). Contrarily, in studies demonstrating the inability of DR5 agonists to induce cardiomyocyte death, DR5 activation was shown to result in ERK1/2 signaling. While ERK1/2 has a well characterized pleiotropic role in the heart (Bueno and Molkentin, 2002), ERK1/2 activation with DR5 agonists occurred through EGFR-transactivation, which has been shown to be protective in cardiomyocytes (Grisanti et al., 2017), and resulted in hypertrophy with mild improvements in contractility, suggesting eccentric hypertrophy. In line with this, in other muscle cell types including smooth muscle cells and skeletal myoblasts, TRAIL also plays a non-canonical role of promoting proliferation or differentiation (Freer-Prokop et al., 2009; van Dijk et al., 2013). However, differences in cardiomyocyte biology may occur during pathological states, which could account for discrepancies between studies. Figure 3 summarizes the known TRAIL and DR5 effects in cardiomyocytes.

### Cardiac fibroblasts

Though studies suggest higher DR5 expression in cardiomyocytes compared with non-myocyte cell populations (Tanner et al., 2019), cardiac fibroblasts express DR5 suggesting a potential role for TRAIL signaling in regulating cardiac fibroblast biology (Tanner and Grisanti, 2021). A panel of human fibroblast cell lines have been shown to express all TRAIL receptors (Crowder et al., 2016). However, the effects of TRAIL on fibroblast populations is poorly understood. Healthy lung fibroblasts are susceptible to TRAIL-mediated apoptosis at high concentrations whereas low concentrations promote collagen production (Yurovsky, 2003) and numerous fibroblast cell lines have been shown to be resistant to TRAIL-induced apoptosis (Crowder et al., 2016). This has been attributed to low caspase 8 protein levels and the inability of fibroblasts to ubiquitinate caspase 8, which is needed for complete activation (Crowder et al., 2016). In these studies, inhibiting deubiquitination made fibroblasts susceptible to TRAIL-induced apoptosis. A single study has examined the role of TRAIL/DR5 in cardiac fibroblasts. Using primary rat neonatal cardiac fibroblasts and adult mouse cardiac fibroblasts from WT and DR5 knockout mice, DR5 activation was found to not induce apoptosis in these primary cell populations. In this study, DR5 activation resulted in proliferation of naïve cardiac fibroblasts. However, upon differentiation of fibroblasts to myofibroblasts with transforming growth factor (TGF)-β, DR5 expression was upregulated and was able to induce apoptosis through the induction of the extrinsic pathway of apoptosis (Tanner and Grisanti, 2021). A chronic β-adrenergic receptor activation model of heart failure in DR5 knockout mice confirmed that more fibrosis occurs in mice lacking DR5. Taken together, these finding suggest that TRAIL and its receptors may help to initiate reparative mechanisms, but decelerate remodeling.

### Other cell populations

The role of TRAIL in inducing immune cell death has been extensively characterized (Sag et al., 2019). In particular, TRAIL-induced apoptosis plays an important function in suppressing inflammation. While reports vary, the general consensus is that neutrophils and monocytes/macrophages express TRAIL under normal conditions whereas most other immune cells do not or have variable expression. However, most immune cell populations can alter TRAIL expression or cleavage of membrane TRAIL to alter circulating TRAIL levels. Additionally, most immune cell populations express DR4/5 and are susceptible to TRAIL-induced death, that varies depending on disease state. While the impact of TRAIL and its receptors in regulating inflammation has not been investigated in the context of the heart, alterations in TRAIL and OPG in PBLs from patients with dilated cardiomyopathy (Schoppet et al., 2005) and TRAIL on PBMCs after myocardial infarction (Nakajima et al., 2003) suggests that immune cells may be an important source of TRAIL in cardiovascular disease. Additionally, regulation of immune responses by TRAIL and its receptors may be an important mechanism of inflammatory control in the heart.

The role of TRAIL and its receptors in endothelial cells is not well described. Human endothelial cells express DR4 and DR5 (Li et al., 2003). Reports on the function of DR4 and DR5 in endothelial cells has been contradictory. Activation by TRAIL resulted in a modest level of apoptosis and in surviving cells, increased cytokine production and adhesion factors that promote inflammation. However, other studies have shown TRAIL reduces inflammation and reactive oxygen species under pro-atherogenic conditions (Forde et al., 2020). TRAIL has also been shown to activate ERK1/2 and Akt in human endothelial cells to promote survival and proliferation (Secchiero et al., 2003). However, the role of TRAIL and its receptors in cardiac endothelial cell function has never been examined.

### TRAIL as a prognostic and diagnostic marker in cardiac disease

Powerful evidence from multiple studies have suggested that TRAIL or soluble DR5 could be a biomarker for the development or progression of cardiac disease. Of particular note, in several prospective observational studies, TRAIL or its receptors were identified as being amongst the most predictive biomarkers identified (Volpato et al., 2011; Hage et al., 2017; Skau et al., 2017; Stenemo et al., 2018). This includes better correlation between TRAIL or soluble TRAIL receptors and cardiac dysfunction or potential to develop severe disease beyond currently used markers such as BNP and CK. Currently, it is impossible to determine what patients will develop severe heart failure or cardiovascular disease. These studies suggest that circulating TRAIL, DR5 or OPG may be a beneficial prognostic tool to predict disease progression and allow for early intervention. However, large scale studies will be needed to confirm these findings in a diverse patient population. Additionally, TRAIL has been implicated in multiple diseases and may be an indicator of inflammatory status or a general disease marker. Further studies will be important in differentiating changes in TRAIL and circulating TRAIL receptors due to cardiac disease from other pathological conditions.

### Targeting TRAIL and its receptors as a therapeutic target in the heart

Studies therapeutically targeting TRAIL/DR5 in the heart are limited. Human data is confounding with some studies suggesting a protective and other pointed towards a detrimental role for TRAIL signaling in the heart. However, administration of soluble DR5 in rats with AMI to prevent TRAIL from binding to functional DRs resulted in reduced infarct size and improved markers of cardiac damage such as serum cardiac troponin I and creatine kinase-MB (CK-MB) (Wang et al., 2023). In a separate study, blocking TRAIL with a soluble DR5 immunoglobulin fusion protein prevented cardiac cell death and inflammation in rats, pigs and monkeys (Wang et al., 2020). However, these studies are contradictory to the majority of human clinical data suggesting low levels of TRAIL are negatively correlated with outcome after myocardial infarction. Furthermore, in a mouse model of diabetic cardiomyopathy, recombinant TRAIL or AAV-mediated TRAIL expression suppressed cardiomyocyte death and cardiac fibrosis. It remains to be seen if inhibiting TRAIL signaling is beneficial in other forms of cardiovascular disease and heart failure from different etiologies. However, the abundance of clinical evidence showing a correlation between cardiac pathology and levels of TRAIL or its receptors would suggest that targeting these mechanisms may be beneficial for the treatment of cardiac diseases. Further understanding of the role of TRAIL and its receptors in cardiac biology will be necessary to determine which receptors are important and how they are function before it will be clear if activating or inhibiting TRAIL receptor signaling is beneficial in the heart. However, the cancer field has paved the way with the development of numerous strategies for therapeutically targeting DR5.

As mentioned, DR5 has been an attractive therapeutic target due to the long-held belief that activation of DR5 specifically induced apoptosis only in transformed cells. Over the past 30 years, numerous approaches have been taken to activate DR5 in the setting of cancer (von Karstedt et al., 2017). This includes clinical trials, which showed no adverse effects in the heart (Ashkenazi et al., 1999; Walczak et al., 1999; Herbst et al., 2010; Soria et al., 2010). Earliest approaches including administering recombinant TRAIL intravenously (Ashkenazi et al., 1999; Herbst et al., 2010). While this is a viable approach for targeting cardiac diseases, repeated administrations are necessary due to the stability and bioavailability of TRAIL. DR5 agonist antibodies have also been a popular approach but have many of the same pitfalls as recombinant TRAIL including poor pharmacokinetics and limited activation of the receptor (Soria et al., 2010). However, this antibody-based approach more specifically targets DR5 whereas recombinant TRAIL administration could affect all TRAIL receptors. TRAIL based approaches could either result in a diluted effect compared with a more specific DR5-targeted approach due to binding to decoy receptors or might be beneficial in the context of the heart if DR5 is not the primary TRAIL effector in the heart. Due to the limited success of these therapies for cancer clinically, further approaches have been taken to attempt to improve the efficacy of these therapies. This includes modified TRAIL to improve its bioavailability, half-life and trimerization however, translation of these therapies has been hindered by the increased potential for toxicity (Di Cristofano et al., 2023). Small molecule therapies are a mainstay in the pharmaceutical industry for their advantages in bioavailability and biodistribution, so it is unsurprising that efforts have been made to develop small molecule DR5 agonists. One small molecule, ONC201, has been shown to induce cancer cell death through the activation of DR5, however, it has been shown to act through the upregulation of TRAIL, thus indirectly causing DR5 activation (Allen et al., 2015). An alternative small molecule, A2C2, has been shown to be a DR5 agonist however, the *in vivo* pharmacokinetic properties have been poorly defined at this time (Wang et al., 2013). Should inhibiting TRAIL/DR5 signaling prove useful in the heart, therapeutic options are more limited. In preclinical rodent models, administration of soluble DR5, which sequesters TRAIL to prevent its binding, has been effective (Wang et al., 2023). In large animal studies, sequestering TRAIL using a soluble DR5 immunoglobulin fusion protein has been used to antagonize DR5 (Wang et al., 2020). Should an inhibitory approach be useful in cardiac diseases, further development and characterization of antagonistic approaches will be necessary.

## Conclusion and perspectives

In the past decade, TRAIL, DR5 and OPG have emerged as important indicators of cardiac diseases. Current understanding of the impact of TRAIL in the heart is limited. While DR4 and DR5 are classically thought to be death-inducing receptors, in non-transformed cell populations, TRAIL often has a non-death-inducing function. Mechanistic studies have not fully defined the role of TRAIL receptor activation and function in relevant cells of the heart. Studies from rodent models show both a pro-apoptotic and beneficial role for DR5 in cardiomyocytes and studies on other cardiac relevant cell populations is limited. Additionally, more is known about the role of OPG in cardiac diseases. However, the affinity of TRAIL for OPG is lower than DR4/5 and it is currently unknown of OPG is playing a role in TRAIL signaling in the heart. Many of these studies focus on the role of OPG in RANKL signaling and fail to consider TRAIL. Furthermore, many studies concerning TRAIL focus on a single receptor and neglect to consider other receptors. Clinical correlations suggest both a protective and detrimental role depending on the study and disease. This may occur in a unique manner depending on the type of cardiovascular disease being investigated. However, it is clear that TRAIL signaling is associated with cardiac disease making further investigation warranted. Better understanding the role of TRAIL and its receptors in cardiovascular disease and how they regulate cardiovascular protection or pathology may offer new diagnostic or therapeutic approaches. Indeed, a limited number of studies have shown a promising role for TRAIL neutralization in rodent, pig and monkey models of myocardial infarction. More careful consideration of how TRAIL is regulating cardiac physiology under normal conditions and during pathophysiology and further mechanistic studies will provide insight into the potential of using TRAIL or its receptors as a prognostic or therapeutic tool in the future.

## Abbreviations

ADAMs, A disintegrin and metalloproteinase; AF, atrial fibrillation; AMI, acute myocardial infarction; BNP, brain natriuretic peptide; CAD, coronary artery disease; CK, creatine kinase; DcR, decoy receptor; DISC, death-inducing signaling complex; DR, death receptor; ERK, extracellular signal-regulated kinase; FADD, fas-associated death domain; GDF, growth differentiation factor; GPI, glyosylphosphatidylinositol; HFpEF, heart failure with preserved ejection fraction; MAPK, mitogen-activated protein kinase; MPPs, matrix metalloproteinases; NF-κB, nuclear factor kappa-B; OPG, osteoprotegerin; PBLs, peripheral blood leukocytes; PBMCs, peripheral blood mononuclear cells; RANK, receptor activator of nuclear factor kappa-B; RANKL, receptor activator of nuclear factor kappa-B ligand; RIP, receptor-interacting protein; TNF, tumor necrosis factor; TRAF, TNF receptor-associated factor 2; TRAIL, tumor necrosis factor related apoptosis inducing ligand.
